# Bioserotypes, Virulence Markers, and Antimicrobial Susceptibility of *Yersinia enterocolitica* Strains Isolated from Free-Living Birds

**DOI:** 10.1155/2020/8936591

**Published:** 2020-03-17

**Authors:** Marta Odyniec, Tomasz Stenzel, Dorota Ławreszuk, Agata Bancerz-Kisiel

**Affiliations:** ^1^Department of Epizootiology, Faculty of Veterinary Medicine, University of Warmia and Mazury in Olsztyn, Poland; ^2^Department of Poultry Diseases, Faculty of Veterinary Medicine, University of Warmia and Mazury in Olsztyn, Poland; ^3^Faculty of Biology and Chemistry, Institute of Biology, University of Białystok, Poland

## Abstract

The risk of meat contamination with *Yersinia enterocolitica* poses a threat to consumers and persons who come into contact with bird carcasses. The occurrence of *Y*. *enterocolitica* in the vast majority of migratory game species, the capercaillie, and the black grouse has never been studied in Poland, Europe, or in the world. The material for the study consisted of cloacal swabs obtained from 143 Eurasian coots, 50 mallards, 30 pochards, 27 greylag geese, 22 white-fronted geese, 22 bean geese, 20 green-winged teals, and 10 tufted ducks, as well as fecal swabs obtained from 105 capercaillie and 18 black grouse. Bacteriological examinations of 894 samples taken from 447 birds led to the isolation of 20 strains with the biochemical features characteristic of the genus *Yersinia*. All 20 strains were molecularly examined, and the genes characteristic of *Y*. *enterocolitica* were detected in 8 strains. The isolated strains harbored amplicons whose size corresponded to *ystB* gene fragments. Four strains belonged to bioserotype 1A/NI, one strain was identified as bioserotype 1B/O:9, and one as 1A/O:9. The prevalence of *Y*. *enterocolitica* was determined at 1.4% in green-winged teals, at 5.0% in Eurasian coots, and at 4.8% in capercaillie. All strains were resistant to amoxicillin with clavulanic acid, ampicillin, and cefalexin. The strains isolated from migratory birds were also resistant to kanamycin and streptomycin, and they were characterized by resistance or intermediate resistance to cefotaxime, ceftazidime, chloramphenicol, gentamycin, and tetracycline, to which the strains isolated from the capercaillie were susceptible. *Yersinia enterocolitica* was not detected in the remaining bird species. The presence of *Y*. *enterocolitica* in green-winged teals, Eurasian coots, and capercaillie indicates that these birds could be carriers, potential reservoirs, and sources of infection for humans. They can also be regarded as reliable bioindicators of *Y*. *enterocolitica* in their respective habitats.

## 1. Introduction

Free-living birds are a highly interesting and insufficiently investigated group of animals. Game birds play an important role in public health as a potential source of infection with pathogens dangerous for human health. In Poland, 13 species of free-living birds can be hunted: pheasant (*Phasianus colchicus*), green-winged teal (*Anas crecca*), tufted duck (*Aythya fuligula*), greylag goose (*Anser anser*), white*-*fronted goose (*Anser albifrons*), bean goose (*Anser fabalis*), pochard (*Aythya ferina*), wood pigeon (*Columba palumbus*), hazel grouse (*Tetrastes bonasia*), mallard (*Anas platyrhynchos*), partridge (*Perdix perdix*), Eurasian coot (*Fulica atra*), and Eurasian woodcock (*Scolopax rusticola*). Most of these birds are migratory species that travel considerable distances during migrations and use natural water resources, which underscores their importance in the epidemiological context. The capercaillie (*Tetrao urogallus*) and the black grouse (*Tetrao tetrix*) are classified as endangered species in Poland [1]. However, despite the progressing decrease in the size of capercaillie and black grouse populations, these species are regarded as game birds in some countries (e.g., Belarus, Russia, Sweden, Finland, and Norway) and are hunted for meat and trophies [1]. Unlike other game birds, the capercaillie and the black grouse do not migrate and inhabit specific territories.

The genus *Yersinia* of the family *Enterobacteriaceae* is composed of 19 species, three of which (*Y*. *pestis*, *Y*. *enterocolitica*, and *Y*. *pseudotuberculosis*) are pathogenic for humans and animals [2, 3]. Although yersiniosis is not highly prevalent and dangerous for animals, animals are often carriers and sources of *Yersinia* infections for humans. In Europe, *Y*. *enterocolitica* is the most important etiological factor of human yersiniosis, whereas *Y*. *pseudotuberculosis* infections rarely cause yersiniosis [4]. Raw or undercooked pork is the main source of human *Y*. *enterocolitica* infections, and pigs are the main reservoir of bacteria [5, 6]. However, *Y*. *enterocolitica* was also detected in various species of wild animals. In Poland, the pathogen has been identified in wild boars, roe deer, red deer, and fallow deer [7–9]. In the group of game birds, only pheasants and mallards have been tested for the presence of *Y*. *enterocolitica* in Poland [10]. The risk of meat contamination with *Y*. *enterocolitica* poses a threat to consumers and persons who come into contact with bird carcasses. The occurrence of *Y*. *enterocolitica* in the vast majority of migratory game species, the capercaillie, and the black grouse has never been studied in Poland, Europe, or in the world.

*Yersinia enterocolitica* strains are classified into six biotypes (1A, 1B, and 2-5) and more than 70 serotypes. Pathogenic strains harbor the *Yersinia* virulence plasmid (pYV) and *ail* and *ystA* chromosomal genes which encode the production of attachment invasion locus (Ail) protein and *Yersinia-*stable toxin A (YstA), respectively [2]. Biotype 1A strains that do not harbor pYV and *ail* and *ystA* genes are usually considered nonpathogenic [2, 11, 12]. However, in recent years, *Y*. *enterocolitica* biotype 1A strains have also been isolated from clinical cases of yersiniosis presenting with diarrhea [6, 13, 14]. Although biotype 1A strains very rarely produce YstA enterotoxin, they usually harbor the *ystB* gene which encodes the production of YstB enterotoxin. The minimal lethal dose of YstB is much lower in comparison with YstA enterotoxin [15, 16]. Extremely rare strains of biotype 1A harbor the *ystC* gene which encodes the production of YstC enterotoxin [16], and they have never been isolated from clinical cases of yersiniosis.

The aim of this study was to identify the bioserotypes, virulence markers, and antimicrobial susceptibility of *Y*. *enterocolitica* strains isolated from the different species of game birds in Poland, as well as from the capercaillie and black grouse.

## 2. Materials and Methods

### 2.1. Materials

The material for the study consisted of 286 cloacal swabs obtained from 143 Eurasian coots, 100 cloacal swabs obtained from 50 mallards, 60 cloacal swabs obtained from 30 pochards, 54 cloacal swabs obtained from 27 greylag geese, 44 cloacal swabs obtained from 22 white-fronted geese, 44 cloacal swabs obtained from 22 bean geese, 40 cloacal swabs obtained from 20 green-winged teals, and 20 cloacal swabs obtained from 10 tufted ducks. Two samples were collected from each bird and cultured in two different media. The samples from free-living birds were collected during the hunting seasons specific for each species, between autumn 2016 and spring 2019, in hunting districts throughout Poland.

Two hundred and ten fecal swabs from 105 capercaillie fecal samples and 36 fecal swabs from 18 black grouse fecal samples were also collected. Most capercaillie were kept as a part of the EU LIFE Project entitled “Active protection of lowland populations of capercaillie in the Bory Dolnośląskie and the Augustowska Primeval Forest”, authorization no. DZP-WG.6401.03.100.2016.km of 31 May 2016, granted by the General Directorate of Environmental Protection. Some samples were collected from wild capercaillie in the Kirov region (Russia) and the Wildlife Park in Kadzidłowo (Poland). All samples were collected from aviaries in breeding centers in 2016-2017. Black grouse samples were collected in June 2017 in the Jedwabno State Forest District which features 5 black grouse preservation zones.

### 2.2. Biochemical Identification

A total of 894 swabs collected from 447 birds were examined. One swab was placed on the ITC (irgasan, ticarcillin, and potassium chlorate—warm culture) medium, and the other swab was placed on the PSB (peptone, sorbitol, and bile salts—cold culture) medium. Swabs on ITC were incubated at 25°C for 48 h, and swabs on PSB were incubated at 4°C for 3 weeks to determine the ability of *Yersinia* to grow at low temperatures. Next, 0.5 ml of each culture was transferred to 4.5 ml of 0.5% KOH in 0.5% NaCl for 20 s, and a loopful was streaked onto a CIN (cefsulodin, irgasan, and novobiocin) plate and incubated at 30°C for 48 h. Colonies typical for *Yersinia* were transferred to MacConkey agar, incubated at 30°C for 24 h and biochemically analyzed using API 20E strips (bioMérieux, France) according to the manufacturer's instructions.

### 2.3. DNA Isolation

Genomic DNA was isolated with the Genomic Mini Kit (A&A Biotechnology, Gdynia, Poland) according to the manufacturer's instructions. DNA was stored at -20°C for further analyses.

### 2.4. Molecular Confirmation of *Y*. *enterocolitica* Strains

Four chromosomal genes—*ail*, *ystA*, *ystB*, and *ystC*—were amplified in one reaction. The primers for *ail*, *ystA*, *ystB*, and *ystC* were described previously [7, 17]. Multiplex PCR was performed using HotStarTaq Plus DNA Polymerase (Qiagen GmbH, Hilden, Germany) and HotStarTaq Plus Master Mix Kit (Qiagen). The reaction mixture of 20 *μ*l contained approximately 120 ng of isolated DNA (1 to 3 *μ*l), 10 *μ*l of the HotStarTaq Plus Master Mix 2x, 2 *μ*l of CoralLoad Concentrate 10x, and 0.1 *μ*l of each primer (final concentration of 0.5 *μ*M), and it was supplemented with up to 20 *μ*l of RNase-free water. The applied reaction conditions included a preliminary denaturation step at 95°C for 5 min, followed by 30 cycles of denaturation at 94°C for 45 s, annealing at 60°C for 60 s, and elongation at 72°C for 45 s. The last reaction was followed by extension at 72°C for 10 min. Amplicon size was evaluated by comparison with the standard mass of GeneRuler 100 bp Ladder Plus (Fermentas UAB, Vilnius, Lithuania). The following amplicons were searched: *ail* gene fragments with the size of 356 bp, *ystA* gene fragments with the size of 134 bp, *ystB* gene fragments with the size of 180 bp, and *ystC* gene fragments with the size of 284 bp. The specificity of all amplicons was confirmed by purification with the CleanUp kit (A&A Biotechnology, Gdynia, Poland) and sequencing (Genomed, Warsaw, Poland).

### 2.5. *Y*. *enterocolitica* Serotype and Biotype Identification

The molecularly confirmed *Y*. *enterocolitica* strains were serotyped in the slide agglutination test according to a previously described methodology [7] with use of somatic antigens O:3, O:5, O:8, O:9, and O:27 (Sifin, Berlin, Germany). The strain was classified as nonidentified (NI) in the absence of agglutination with any of the sera. The biotype was determined based on pyrazinamidase and Tween esterase activity, esculin hydrolysis, indole production, and salicin, xylose, and trehalose fermentation according to the PN-EN ISO 10273 standard.

### 2.6. Antimicrobial Susceptibility of the Isolated *Y*. *enterocolitica* Strains

*Yersinia enterocolitica* strains were tested for susceptibility to amoxicillin with 30 *μ*g of clavulanic acid, 10 *μ*g of ampicillin, 30 *μ*g of cefotaxime, 30 *μ*g of ceftazidime, 30 *μ*g of cefalexin, 5 *μ*g of ciprofloxacin, 30 *μ*g of chloramphenicol, 10 *μ*g of gentamycin, 30 *μ*g of kanamycin, 30 *μ*g of nalidixic acid, 10 *μ*g of streptomycin, sulfamethoxazole/trimethoprim 19 : 1, and 30 *μ*g of tetracycline (Oxoid, Thermo Scientific). These analyses were performed by the standard disc diffusion technique after 24 h incubation on Mueller-Hinton (Oxoid, Thermo Scientific) agar plates at 30°C, and they were interpreted according to Clinical and Laboratory Standards Institute (CLSI) guidelines [18].

## 3. Results

Bacteriological examinations of 894 samples collected from 447 birds led to the isolation of 20 strains with biochemical features characteristic of the genus *Yersinia*. Seven strains were classified as *Y*. *kristensenii*, 5 strains as *Y*. *frederiksenii*, 7 strains as *Y*. *enterocolitica*, and 1 strain as *Y*. *frederiksenii*/*Y*. *intermedia* with the use of API 20E. All 20 strains were molecularly examined, and the genes characteristic of *Y*. *enterocolitica* were detected in 8 strains. Six strains were biochemically identified as *Y*. *enterocolitica*, and the remaining two strains were identified as *Y*. *frederiksenii*. One strain, initially classified as a *Y*. *enterocolitica* with API 20E, was not molecularly confirmed.

The prevalence of *Y*. *enterocolitica* in green-winged teals was determined at 1.4%. Two strains were isolated from the samples collected in the Małopolska voivodeship. The strains harboring amplicons of *ystB* gene fragments originated from the warm culture (ITC). They belonged to biotype 1A and were serotyped as NI (nonidentified) due to the absence of agglutination with any of the analyzed sera. The results of the biochemical and molecular analyses and the biotypes and serotypes of the isolated *Y*. *enterocolitica* strains are presented in Table 1.

All *Y*. *enterocolitica* strains were resistant to amoxicillin with clavulanic acid, ampicillin, cefalexin, kanamycin, and streptomycin. They were intermediately resistant to cefotaxime, ceftazidime, chloramphenicol, and tetracycline. All *Y*. *enterocolitica* strains were susceptible only to sulfamethoxazole with trimethoprim, and they differed in susceptibility to the remaining antimicrobials. The antimicrobial susceptibility of *Y*. *enterocolitica* strains is presented in Table 2.

The prevalence of *Y*. *enterocolitica* in Eurasian coots was determined at 5.0%. Similarly to green-winged teals, one strain was isolated from the sample collected in the Małopolska voivodeship. This strain was *ystB*-positive, and it originated from the cold culture (PSB) and was bioserotyped as 1A/NI (Table 1). The antimicrobial susceptibility test revealed that the isolated *Y*. *enterocolitica* strain was resistant to amoxicillin with clavulanic acid, ampicillin, cefalexin, cefotaxime, kanamycin, and streptomycin. The strain was susceptible to nalidixic acid and sulfamethoxazole with trimethoprim, and it was intermediately susceptible to the remaining antibiotics. Detailed characteristics are presented in Table 2.

The prevalence of *Y*. *enterocolitica* in capercaillie fecal samples was determined at 4.8%. Five strains were isolated from the fecal material collected in the Wildlife Park in Kadzidłowo, and the prevalence of *Y*. *enterocolitica* in these samples reached 62.5%. All strains harbored amplicons whose size corresponded to *ystB* gene fragments. Three strains originated from the ITC culture, and 2 strains originated from the PSB culture. Four strains belonged to biotype 1A, and only one strain was identified as biotype 1B. Serotyping revealed that 1 of the 5 isolated *Y*. *enterocolitica* strains represented the O:9 serotype, and the remaining strains were not identified. Two *Y*. *enterocolitica* strains with different bioserotypes (1A/O:9 and 1A/NI) were isolated from a fecal sample collected from the same female. Detailed results of the biochemical and molecular analyses and the biotypes and serotypes of the isolated *Y*. *enterocolitica* strains are presented in Table 1. All *Y*. *enterocolitica* strains were resistant to amoxicillin with clavulanic acid, ampicillin, and cefalexin. One strain was additionally resistant to sulfamethoxazole with trimethoprim, and one strain was also resistant to kanamycin. Two of the tested strains were intermediately resistant to chloramphenicol. All *Y*. *enterocolitica* strains were susceptible to the remaining antibiotics. The antimicrobial susceptibility of *Y*. *enterocolitica* strains is presented in Table 2.

The bacteriological examinations of 36 fecal swabs from black grouse led to the isolation of 1 strain with biochemical features characteristic of the genus *Yersinia*. This strain was biochemically classified as *Y*. *kristensenii*. The molecular analysis confirmed that the strain did not harbor amplicons of *yst* genes characteristic of *Y*. *enterocolitica*.

One strain with biochemical features characteristic of the genus *Yersinia* was also isolated from white-fronted geese. The molecular analysis confirmed that this strain harbored amplicons of the *ail* gene. However, the *ail* gene never occurs alone in *Y*. *enterocolitica* strains, and it is always accompanied by one of the *yst* genes. In biochemical tests involving API 20E, this strain revealed features characteristic of *Y*. *frederiksenii*/*Y*. *intermedia*.

Strains with biochemical features characteristic of the genus *Yersinia* were not identified in bacteriological analyses of the samples collected from mallards, pochards, greylag geese, bean geese, and tufted ducks.

## 4. Discussion

*Yersinia enterocolitica* is one of the most important etiological factors of human diarrhea [4]. Research into the prevalence of *Y*. *enterocolitica* in various animal species is vital because products of animal origin are the main sources of infection for humans [19]. The prevalence of *Y*. *enterocolitica* in wild animals has been studied in Poland, Europe, and in other regions of the world. However, some species of free-living birds have never been investigated. In Poland, mallards and pheasant were the only bird species to be tested for *Y*. *enterocolitica*. Molecularly confirmed *Y*. *enterocolitica* strains were isolated from 5 out of 45 mallards (11.1%) and were not detected in any of the tested pheasants [7].

In Europe, Levrè et al. [20] examined intestinal loops from 217 birds of 17 species. Thirty-seven strains belonging to the genus *Yersinia* were isolated from 26 birds. Most of the isolates were identified as *Y*. *enterocolitica* (28 strains), 3 isolates as *Y*. *frederiksenii*, 3 isolates as *Y*. *intermedia*, and 1 isolate as *Y*. *pseudotuberculosis*. However, molecular tests were not conducted. More recently, Foti et al. [21] tested 218 fecal swabs and 21 internal organs from different migratory birds. Eight *Y*. *enterocolitica* strains (3.35%) were detected in analyses involving bacteriological examinations and API 20E, but molecular tests for *Y*. *enterocolitica* were not carried out.

The prevalence of *Y*. *enterocolitica* in wild birds was also studied outside Europe. In Japan, Kato et al. [22] detected *Y*. *enterocolitica* strains in 2 pheasants, 1 domestic pigeon, 1 tree sparrow, 5 crows, 5 blue magpies, 4 bulbuls, and 2 grey starlings, which accounted for 4% of the tested samples. Shayegani et al. [23] found that 3.3% of 576 wild birds examined in the USA were positive for *Y*. *enterocolitica*. Bacterial strains were isolated from 2 great horned owls, 1 Canada goose, 2 wild turkeys, 1 mallard, 1 red-tailed hawk, 1 cowbird, 1 starling, 2 grackles, 1 ring-billed gull, 1 ruffed grouse, 2 red-winged blackbirds, 1 sparrow hawk, 1 canvasback duck, 1 long-eared owl, and 1 wood duck. In Malaysia, *Y*. *enterocolitica* strains were isolated from 32 out of 291 (11.0%) examined wild ducks and from 13 out of 180 (7.2%) examined wild geese [24].

In our study, *Y*. *enterocolitica* strains were detected in 1.4% of green-winged teals, 4.8% of capercaillie, and 5.0% of Eurasian coots. Our findings do not differ significantly from those reported by other authors, but none of the cited authors relied on molecular methods which are much more accurate and reliable. In our study, the results of the analysis conducted with API 20E did not fully correspond to the results of the molecular test for *Y*. *enterocolitica* virulence markers. Four strains were identified as *Y*. *enterocolitica* using API 20E, but *ystB* gene fragments were detected in 3 strains only. However, 2 strains identified as *Y*. *frederiksenii* with API 20E were molecularly classified as *Y*. *enterocolitica*. The above findings indicate that molecular examinations are essential for proper detection and classification of the isolated strains.

Interestingly, the strains identified in the capercaillie were isolated from a single location. The above could be attributed to the fact that *Y*. *enterocolitica* is easily transmitted. The pathogen is transferred between animals when the carriers are present in the environment [25]. The high percentage of *Y*. *enterocolitica* strains isolated in the Wildlife Park in Kadzidłowo could result from pathogen transmission in aviaries in breeding centers. The above could pose a threat for humans who come into direct contact with infected birds.

In our study, 1A/NI was the predominant bioserotype. The presence of single *Y*. *enterocolitica* strains belonging to bioserotypes 1A/O:9 and 1B/NI was also demonstrated. In a study by Kato et al. [22], *Y*. *enterocolitica* strains were also classified as biovar 1 (biotype 1) with different serotypes (O:3; O:4; O:4,32; O:5A; O:6,30; O:7,8; and O:14). Shayegani et al. [23] classified 12 out of 19 *Y*. *enterocolitica* strains into nine serogroups (O:4,33; O:5,27; O:6,31; O:7,14; O:8,14; O:16; O:30,31; O:31; and O:34), and the remaining strains were not identified. In our study, 2 *Y*. *enterocolitica* strains with different bioserotypes were isolated from one fecal sample. Nikolova et al. [26] isolated different *Y*. *enterocolitica* strains from the same animal, which could indicate that the gastrointestinal tract can be colonized by more than one type of *Y*. *enterocolitica* strains.

The antimicrobial susceptibility analysis revealed that all tested isolates were resistant to amoxicillin with clavulanic acid, ampicillin, and cefalexin. It should also be noted that the strains isolated from migratory birds (green-winged teals, Eurasian coots) and those obtained from birds living in a specific territory (capercaillie) differed in resistance to chemotherapeutics. The strains isolated from migratory birds were also resistant to kanamycin and streptomycin, and they were resistant and intermediately resistant to cefotaxime, ceftazidime, chloramphenicol, gentamycin, and tetracycline, to which the strains isolated from the capercaillie were susceptible. The fact that these strains were susceptible to only one or two chemotherapeutics gives serious cause for concern because migratory birds use public drinking water sources. Our results cannot be compared with the findings of Foti et al. [21] who studied antimicrobial susceptibility but did not report specific results for *Y*. *enterocolitica* strains.

## 5. Conclusions

The presence of *Y*. *enterocolitica* in green-winged teals, Eurasian coots, and capercaillie indicates that these birds could be carriers, potential reservoirs, and sources of infection for humans. These birds can also be regarded as reliable bioindicators of *Y*. *enterocolitica* in their respective habitats.

## Figures and Tables

**Table 1:** Characteristics of *Y*. *enterocolitica* strains isolated from free-living birds.

Strain no.	Bird species	Biochemical identification	Molecular examination				Molecular identification	Bioserotype
		API 20E	*ail*	*ystA*	*ystB*	*ystC*		
30PSB	Capercaillie	*Y*. *enterocolitica*	-	-	+	-	*Y*. *enterocolitica*	1A/NI
31PSB	Capercaillie	*Y*. *frederiksenii*	-	-	+	-	*Y*. *enterocolitica*	1A/NI
35ITC	Capercaillie	*Y*. *frederiksenii*	-	-	+	-	*Y*. *enterocolitica*	1A/NI
35PSB	Capercaillie	*Y*. *enterocolitica*	-	-	+	-	*Y*. *enterocolitica*	1A/O:9
36ITC	Capercaillie	*Y*. *enterocolitica*	-	-	+	-	*Y*. *enterocolitica*	1B/NI
116ITC	Eurasian coot	*Y*. *enterocolitica*	-	-	+	-	*Y*. *enterocolitica*	1A/NI
117ITC	Eurasian coot	*Y*. *enterocolitica*	-	-	+	-	*Y*. *enterocolitica*	1A/NI
1GWPB	Green-winged teal	*Y*. *enterocolitica*	-	-	+	-	*Y*. *enterocolitica*	1A/NI

**Table 2:** Antimicrobial susceptibility of *Y*. *enterocolitica* strains isolated from free-living birds.

Antimicrobial	Strain no.							
	30 PSB	31 PSB	35 ITC	35 PSB	36 ITC	116 ITC	117 ITC	1GW PSB
Amoxicillin+clavulanic acid 30 *μ*g	R^a^	R	R	R	R	R	R	R
Ampicillin 10 *μ*g	R	R	R	R	R	R	R	R
Cefotaxime 30 *μ*g	S^b^	S	S	S	S	I	I	R
Ceftazidime 30 *μ*g	S	S	S	S	S	I	I	I
Cephalexin 30 *μ*g	R	R	R	R	R	R	R	R
Ciprofloxacin 5 *μ*g	S	S	S	S	S	I	S	I
Chloramphenicol 30 *μ*g	I^c^	S	S	I	S	I	I	I
Gentamycin 10 *μ*g	S	S	S	S	S	R	I	I
Kanamycin 30 *μ*g	S	R	S	S	S	R	R	R
Nalidixic acid 30 *μ*g	S	S	S	S	S	R	I	S
Streptomycin 10 *μ*g	S	S	S	S	S	R	R	R
Sulfamethoxazole/trimethoprim 19 : 1	R	S	S	S	S	S	S	S
Tetracycline 30 *μ*g	S	S	S	S	S	I	I	I

^a^R: resistant; ^b^S: susceptible; ^c^I: intermediately resistant.

## Data Availability

The data used to support the findings of this study are available from the corresponding author upon request.
